# The Pathological Anatomy and Pathogenesis of Disseminated Chronic Pneumonia, and of Pulmonary Tubercles

**Published:** 1868-09-01

**Authors:** H. Lebert

**Affiliations:** Professor of Clinical Medicine of the University of Breslau


CHICAGO MEDICAL JOURNAL.

*Vol. XXV.--SEPTEMBER* 1, 1868.—*No.* 17.

### PATHOGENESIS.

### *The Pathological Anatomy and Pathogenesis of Disseminated Chronic Pneumonia, and of Pulmonary Tubercles. By* H. LEBERT, *Professor of Clinical Medicine of the University of Breslau.*

TRANSLATED BY WALTER HAY, M.D., ASSOCIATE EDITOR CHICAGO MEDICAL JOURNAL.

IN the following pages I shall publish a few of my lessons upon chronic pneumonia and pulmonary tubercles. I should have attempted to give them entire, but that such a work would transcend the limits of an article for a periodical.

It is well known that for a long time the study of the diseases of the respiratory passages has possessed for me an especial attraction. Engaged for quite a long period in the experimental production of inflammatory and tubercular alterations in the lungs, I hope soon to be able to assemble all my experiments in a special work upon this important subject; but I desire at present to give a *resumé* of my anatomical and pathogenetic experiments upon this subject, seeing that, at this moment, it occupies the attention of pathological anatomists as well as of clinical observers, and indeed, that there is no subject of greater importance, since it concerns diseases which harvest nearly a fifth part of the human race. The thorough investigation of the mode of existence, and of the formation of such maladies, must, sooner or later, result in effecting a diminution of the ravages of this terrible pestilence.

Before commencing the anatomical description, I should say that I avoid throughout this work, as much as possible, the term "phthisis pulmonalis," especially as it preceded the study of tubercle, and as it appears to have survived the unity of tuberculosis. It would indeed be convenient to understand by this term disseminated chronic pneumonia and pneumophymia (?), associated by a strong affinity, although all recent researches protest against their identity. Indeed, true tuberculosed subjects may succumb after an acute or subacute attack, with figures yet athletic, and with still a sub-cutaneous layer of fat. On the other hand, disseminated chronic pneumonia, in its mild and slightly febrile form, often permits its subjects, who have already cavities in their lungs, to occupy themselves with their business with a degree of vigor and a rotundity of figure far different from that of phthisis. Moreover, the patients who lose flesh and strength under, during the subacute exacerbations of disseminated pneumonia, recover them considerably, when the progress of the malady is retarded and the fever ceases, and the same subjects again emaciate when new foci of disease develop themselves. Thus a patient may be phthisical to-day, be so no longer in three months, and again be so in six; moreover, do not the subjects of chronic catarrh and bronchial dilatation become emaciated by a prolonged and considerable loss of albuminoid substance? Phthisis is, therefore, a morbid effect, and not a disease.

Another term, also very generally employed, and likewise entirely irrational, is that of the "caseous state." Nothing less resembles cheese, under its relations of structure, chemical composition and exterior aspect than the yellow infiltrations termed "caseous;" I am astonished that physicians, lovers of cheese, should not have long since protested against this term, which the lovers of correct and exact medical nomenclature ought very thoroughly to reprobate. To speak of an infiltration as gray, yellow, circumscribed or diffuse, gives in truth a much clearer idea of the morbid tissue than to designate it as "cheesy." I avoid, with equal care also, the term scrofulous pneumonia. Scrofula, in the majority of cases, having nothing to do with pneumonia. The foci do not correspond with the limits of the lobules, their existence being just as probable in the interstitial and peribronchial connective tissue as in the alveoli. I prefer, likewise, the term disseminated pneumonia to that of lobular, or broncho-pneumonia.

The preliminary remarks concluded, we proceed to the anatomical descriptions.

### I.—Pathological Anatomy.

We shall successively pass in review chronic disseminated pneumonia, originating from mechanical causes; then that developed spontaneously, and then true pulmonary tubercles.

We shall disregard chronic diffused and lobular pneumonia, which we have likewise observed and studied anatomically a sufficient number of times, and which is far from being so rare as some authors assert. However, its anatomical and clinical characteristics may associate it more closely with acute or subacute pneumonia, than with the little disseminated foci, or with tubercles, yet both these conditions may be fonnd combined with diffuse infiltration.

1. *Chronic Disseminated Pneumonia from mechanical causes.*—The physicians who are engaged in the investigation of the subject of pulmonary tubercles, and of all relating to them, have very generally neglected the study of pulmonary inflammations from mechanical causes, because they believed them not to be correlated with tubercle. To-day, since the unity of chronic diseases of the lungs no longer exists, the majority of cases, considered formerly as tuberculous, but of slow progress, are shown to be, in reality, foci of inflammatory origin, the study of an intricate inflammatory process could not fail to be benefitted by comparison with similar foci, having an origin very well understood.

We have referred, in a preceding lecture, to the enormous mortality from maladies of the chest, observed by Peacock, of London, and Lewin, of Berlin, amongst the dressers of stone, especially of hard stones and mill stones. Lewin has recognized sharp and pointed bony particles in the expectoration of these laborers. Although anatomical investigations upon this subject are not numerous, yet Bristowe has discovered, from an observation of Peacock, in one of these lungs a very considerable quantity of cutting particles, sharp and transparent, of those collected in the workshop, composed of quartz and of silica.

We are in possession of very exact investigations of Zunker upon diseases of the lungs, caused by ferruginous particles. He has discovered *Oxyde of Iron* in all portions of the lungs, even in the pleura and bronchial glands, as also in the epithelial cells of the ultimate ramifications of the bronchial tubes, and of the air-cells. Many particles of iron having traversed the cellular parietes had reached the pulmonary tissue, especially in the lobular and fundibular partitions, as well as in the sheaths of the bronchial tubes, the sub-pleural tissue, and thence into the pleura, even traversing the lymphatic vessels. The iron had penetrated, not only into the bronchial glands, but also in those which are found by the side of the trachea. These serous (?) fragments had produced interstitial granulations, callous indurations corresponding to the lobules, like a pneumonia, disseminated, lobular, interstitial and peribronchial. There were, moreover, vomicæ, originating, probably, from bronchial ulcerations, and at the surface of the indurated portions of the partial pleuritic thickening.

Hence it appears that granules of iron, produce, eventually, in the lungs, all the manifestations, all the forms, all the phases of disseminated chronic pneumonia.

Coal-dust is less irritating to the lungs, and when it reaches them in small quantities, it appears even salutary in those occupations where it is used in moderate quantities. This is, however, not the case with workmen in mines of pit-coal, and there has been observed, during a long period, the state of anæmia, and the impeded and prolonged respiration with which those who have labored for a long time in those mines are so frequently troubled whilst the lungs are healthy, and there is no chronic catarrrh, no trouble supervenes, and even in cases of bronchitis, the vibratile movements in the bronchial tubes for a long time prevents the particles of coal depositing themselves in the cells; a black expectoration accompanies the pulmonary catarrh of the laborer, and Traube has demonstrated that this dust has penetrated into the cellular elements of the expectoration.

But, at a later period these fragments of coal would reach the cells, pass through them, and forment at first, in the pulmonary parenchyma, little striated strains, which, at a later period become thicker, more compact, more confluent. It is only exceptionally that these masses attain to the size of a nut. They are found more frequently in the lower and middle lobes ahan in the upper. Masses are found also in some of the terminal bronchial ramifications, thence by degrees the bronchial glands become infiltrated. Whenever a chronic catarrh, with pulmonary emphysema is established, these alterations, at first very slow in developing themselves, are multiplied, and become more considerable. They terminate by constituting themselves true foci of disseminated chronic pneumonia, on this occasion not resembling the cheese of classic authors, but rather their fuel. These foci tend, at a later period, to a destructive process. It is thus that the carbonaceous cavities are formed, which, however, are far from being frequent, and rarely attain the dimensions of pneumonic cavities; their walls are irregular, sinuous, surrounded with a blackish compact tissue; they are met with as well in the inferior as in the superior lobes, in the centre as at the periphery. It is not unusual to find recent or old pleurisy, in its plastic pseudo-membranous form, originating from superficial carbonaceous foci. In rare cases there are found little abscesses in the vicinity of the indurations and the black cavities. It is only under exceptional circumstances that true tubercles are found in these lungs.

The more abundant the silica contained in this carbonaceous dust, the more rapid and invasive will be the progress of these alterations.

2. *Chronic Disseminated Pneumonia without mechanical causes.*—I believe that I do not go too far in saying that the greater portion of the cases regarded as chronic tuberculous affections belong to this disease, with these characteristic alterations.

The most frequent anatomical form is alveolar inflammation, alveolitis. In these cases are found foci, from yellow granulations of very small size, miliary like true tubercles up to larger infiltrations, lobular, more voluminous yet, and confluent. There are found, moreover, peribronchial foci, with very considerable proliferation of cellular connective tissue around a bronchus, which often encloses in its cavity a thick, yellowish, cellular mass, easily detached. Finally, there are formed, more or less, considerable interstitial foci, from the size of a pea up to that of a small almond, composed entirely of the budding out and multiplication of the cells of the connective tissue. To constitute out of these diverse localizations particular species, would be so much the more unreasonable, as they are found combined together in almost every manner; but the proliferation of connective tissue, playing here as important a part as that of the epithelial. I am unwilling to accept the term epithelial pneumonia employed by some authors. The expression involves truth, but too partial to permits its application to all these foci, cellular (*alveolaires*), peribronchial and interstitial. In order to avoid an exclusive interpretation, I should say that true tubercles may be the point of departure of these foci; but this is the exception, not the rule. It is much more frequent to find tubercular granulations, disseminated either around pneumonic foci, or in other parts of the lungs.

I believe it may be established as an invariable rule, that when the two orders of alterations are found together, the oldest and most advanced pneumonic foci have been the primitive affection, and the tuberculous granulations have been developed there in a consecutive and secondary manner. Very often, also, disseminated chronic pneumonia exists without trace of tubercle.

When these disseminated chronic pneumonias are examined, either in the condition of yellow granulations, or as more extensive circumscribed infiltrations, the microscope gives no satisfactory result. The proliferation of connective tissue, both interstitial and peribronchial, exhibits not elements resembling in all respects those of true tubercle; but these are found, moreover, here and there, under the form of granulations, which would be supposed, at first sight, to be tuberculous, if they were not found involved in the midst of irritation of connective tissue of diffuse interstitial pneumonia. When these foci have for their point of origin the air-cells, groups of cells and the lobules, there are found, especially towards the centre, indistinct, shriveled cells, resembling very closely the elements of tubercle. It is true that in the peripheral layers there are observed cells of pus and epithelium—these last swollen and granular, as I have described them from my former observations, and as they are likewise often encumbered around true tubercular granulations, may be either gray or yellowish. Indeed, it is this fact which has induced me for a long time to suspect the difference between pneumonic foci and true tubercle. I must admit that formerly my investigations upon this subject did not take into consideration sufficiently the formation, the generation of these products. On the other hand, the methods of investigation were too imperfect at that time, and, moreover, I recognized, since 1842, vesicular pneumonia, having previously served in L'Hôpital des Enfants with Messrs. Legendre and Bailly, whose investigations into the subject are justly valued even to-day. It is true, that, like them, I, for a long time, recognized only the acute form of alveolitis. However, in 1851, I presented to the anatomical society the lungs of a dog, into whose jugular vein I had injected mercury, and in which I had been able to determine the development of purulent foci, at once vesicular and lobular.

Without regarding more antiquated opinions, Reinhardt, an equally good pathological anatomist and micrograph, had long since demonstrated that solid, disseminated foci of a tuberculous appearance in the lungs, were of an inflammatory character; still, he did not take sufficient account of true tubercle, whilst Virchow, making use of what was true in the investigations of Reinhardt, separated true tubercles from these disseminated pneumonias, which he designated as caseous or scrofulous. Other distinguished authors have employed themselves upon this subject in France, especially Villemin, Herard and Corme, etc. In Germany, L. Meyer and others; but no one has better described the formation of these inflammatory products of chronic pneumonia than Colberg,* who had admirable methods of investigation. Already, previous to the recent publication of his memoir, I had commenced, with Dr. Nyss, the chief of my laboratory, experiments upon the inoculation of tubercle and other morbid products, and we have together revised my old experiments upon artificial irritation of the respiratory passages. Moreover, after the manner of Colberg, we have injected the majority of the lungs which we wished to examine, and made use of all the modern appliances for microscopic preparations. We have preserved a sufficiently large number in our collection of pathological anatomy in our laboratory, to enable us to supply a demonstration. Indeed, in order to decide difficult and arduous questions, it is necessary to examine, in a fresh state, but also after having been hardened and submitted to different liquids, the thin, injected slices, in order to be able to discover and to distinguish that, which, whether of tubercle or inflammatory product, takes its origin from the capillaries, from the air-cells, from the small bronchi, from the interstitial tissue, etc.

But one should not be intimidated by the length and difficulty of these investigations. After having made them a certain number of times, it is possible to distinguish with the naked eye all the varieties of pulmonary alterations, which

* Deutches Archiv. fur Clinische Medicin. 1866. 2 Band. 4 a 5 Heft, p. 153.

we have already done, up to the present time, in the presence of pupils, whilst practicing autopsies.

The study of acute and subacute vesicular pneumonia, enables us to see the alveoli (air-cells), filled with muco-pus with epithelial and pus cells, more extensive purulent cavities, even to little foci of lobular hepatization, with a granular, yellowish section. In all these conditions, the hyperplastic product of the epithelium, whether alveolar (cellular), or especially bronchial, may become thickened, and solidify itself, so to speak. In disseminated chronic pneumonia, the smaller foci of a dead yellow, may vary from the size of a grain of dust to that of a hemp-seed, and larger, forming thus miliary granulations, but more dead, more yellow, more friable. These yellow granulations may group themselves together and form little infiltrations from the size of a lintel to that of a pea, or larger, yellow with a smooth and dry section, which, generally, from the very smallest, are scattered in a different manner, without clearly defined limits, in the surrounding pulmonary tissue. Already amongst these infiltrations, still small, it is not unusual to find in the centre, a little opening, corresponding to a lobular bronchus. In places, these masses exhibit a grayish color, probably from the remains of the capillaries which have disappeared, very generally the injections penetrated sufficiently far around these little inflammatory foci, without at all reaching into their interior.

In proportion as the granulations and lobular infiltrations are multiplied and become more confluent, infiltrations are developed, more extended, rounded, lobular, exhibiting a superficies like an oak leaf: a condition very well represented in the designs of Messrs. Herard and Cornel. It is not unusual to find around the miliary alveolar deposits, and between the confluent infiltrations a substance of a deep or reddish gray, partially transparent, which, in a more advanced state becomes firmer, more deep, and assumes a callous aspect of a slaty or blackish gray, in consequence of a considerable quantity of pigment. The infiltrated substance of a reddish gray originates often from immature hyperplasia of the interstitial pulmonary connective issue. It may also exist under the form of little circumscribed masses; most frequently they are found combined with the yellowish foci of cellular (alveolar) origin. We are already familiar with inflammation around the bronchial tubes. More frequently still it is found around the larger bronchial tubes. These peribronchial foci have, at first, a grayish aspect, succulent, and are then composed of the cellular elements of the connective tissue in process of proliferation, and even in the midst of this diffuse inflammation are found some grayish granulations which resemble true tubercles. Later, these buddings of connective tissue become more yellow, drier; the bronchus, surrounded by this tissue, is ordinarily filled with the thickened product of its own secretion easily separable. Hence foci, miliary, lobular, confluent, extended, foci alveolar, peribronchial and interstitial, combined in every manner, but with a predominance of alveolar inflammation, constitute the anatomical elements of disseminated chronic pneumonia.

We have already seen in what manner the interstitial, peribronchial, inflammatory products are formed; as to those of the cells, they are certainly seen at an early period filled with very numerous epithelial cells in process of proliferation, as well as numerous cells of pus, either free or enclosed in the interior of the cells.

This multiplication of epithelia and formation of purulent elements, constitute the anatomical elements of alveolitis, without considering the hyperæmia of the capillary vessels around the alveoli; but the great difficulty is to determine their point of departure. Colberg, who has so well described their genesis, asserts that they originate from the continuous epithelium of the alveoli. It is known that among micrographs some recognize the existence of this alveolar epithelium, whilst others deny it. I have endeavored in vain to detect it in the adult man, even in preparations injected and dried, and in fine sections in various directions. I have seen there only intercapillary cells, such as Villemin and others describe. Now, these cells exist in too small numbers to explain those so considerable cellular masses. I should, therefore, be much more disposed to believe, as in lobular pneumonia, in the hyperplasia of the epithelium of the ultimate bronchial terminations, budding out, multiplying themselves towards the alveoli, and terminating by filling them. Nevertheless, the question is still undecided, and very difficult to resolve. These epithelial elements form not only islets of alveolar infiltration, but also yellow, miliary granulations. These cells speedily undergo a profound alteration by granular or granulo-fatty infiltration, which render their appearance more opaque, then their interstitial serum is quickly absorbed; which, then, gives to the section an aspect dry, and of a dead yellow (caseous of authors). The capillaries not penetrating them, it is easy to understand the retrograde changes, cellular death, the "necrocythosis" of the inflammatory elements.

The debris of the capillaries form pigmentary granulations and stripes, grayish or darker, are abundant around the foci. Once the shells of the shrivelled alveoli becoming granular the resemblance to yellow tubercle is great, whilst their irregular diffusion in the pulmonary tissue, and their mode of formation render them easily distinguishable from true tubercle. Moreover, alveolitis involves as well the interstitial and peribronchial cellular tissue, whilst, on the other hand, inflammation of the connective tissue is combined with that of the alveolæ. It is not right, therefore, to give to chronic disseminated pneumonia the name of epithelial, a term proposed by several authors, but which would be too exclusive. When the inflammatory foci exist in good numbers, with a good state of the general health, they may gradually disappear completely, or diminish in volume, forming yellowish masses resembling glaziers' putty, or constituting little callous indurations of the cellular tissue, in which a species of stony mortar remains as a residue of the alveolar foci. These diverse conditions are combined in every mode, and are found by preference at the summit of one or both lungs, frequently even with a retraction of a cicatricial appearance on the surface. Sometimes, also, there are found in their neighborhood thickened branchlets, obliterated or filled with the concrete product of their secretion.

It is not unfrequent that epithelial or purulent hyperplasia, exhausts itself speedily in the alveoli; and then it is the connective elements of the neighborhood which furnish the ulterior purulent elements.

The considerable masses of cellular elements described, excite in their turn an irritative process in their neighborhood. All these products tend to disintegration; a species of molecular necrosis progresses step by step, and it is thus that ulcerations are formed, at first small, then more and more voluminous, sometimes confluent. Ulcers known under the name of pulmonary caverns (*vomicæ*), surrounded often by more recent foci, which, in their turn, may undergo the same alteration. These irregular cavities tend to invest themselves with a membrane, which, when it covers them completely, may isolate them, and contribute gradually to their cicatrization; but most frequently the contents of the cavity are muco-purulent, and enclose the detritus of all these tissues in process of decomposition, mingled sometimes with the mycelia of fungi. The branches which open into these caverns are ordinarily ulcerated, and, as it were, cut perpendicularly at their opening, whilst the bronchial dilatations, equally frequent in the summit of lungs attacked by chronic pneumonia, are distinguished by the continuity of the mucous membrane of the branches into the whole of the dilated cavity. It is not unfrequent to see in these cavities larger blood-vessels which traverse them, and whose rupture produced the cavernous hæmorrhage, sometimes speedily fatal. The walls enclose also numerous capillaries, and their hair-like appearance is especially manifest when their cavities are examined with the lens under water. In fortunate cases, the inflammation of the connective tissue forms around the pulmonary ulcer a thick, callous substance, which, by becoming more and more dense, may induce from without inward the retraction and final cicatrization from the cavity. All these ulcers, from the very smallest, may, when they are situated svperficially, open into the pleura, a perforation known under the title pneumo-thorax, and in rare cases with external perforation through the thoracic walls with pleuro-pulmonary fistula.

(*To be continued.*)

